# Spurious Plasma Adrenocorticotropic Hormone Level in a Patient With Adrenal Cushing Syndrome

**DOI:** 10.31486/toj.25.0093

**Published:** 2026

**Authors:** Sarath Chandran, Alpesh Goyal, Mangesh Mane, Rahul Gupta, Akansha Choudhary

**Affiliations:** ^1^ Department of Endocrinology and Metabolism, All India Institute of Medical Sciences, Bhopal, India; ^2^ Department of Medical Oncology and Hematology, All India Institute of Medical Sciences, Bhopal, India

**Keywords:** *Adrenocortical carcinoma*, *adrenocorticotropic hormone*, *Cushing syndrome*, *diagnostic errors*, *immunoassay interference*

## Abstract

**Background:**

Endogenous Cushing syndrome results from excessive cortisol production by the adrenal glands and is characterized by the loss of circadian rhythm of cortisol secretion and negative feedback regulation of the hypothalamic-pituitary-adrenal axis. This condition is broadly subtyped into adrenocorticotropic hormone (ACTH)–dependent and ACTH-independent types based on ACTH measurement. A plasma ACTH level <5 pg/mL suggests ACTH-independent Cushing syndrome (adrenal source), while a level >15 pg/mL suggests ACTH-dependent Cushing syndrome (pituitary or ectopic source). A certain immunoassay platform (Immulite 1000 [Siemens AG]) has a known positive ACTH bias, so a patient with ACTH-independent Cushing syndrome may be falsely labeled as having ACTH-dependent disease. This misclassification can lead to unnecessary investigations and interventions such as pituitary imaging, inferior petrosal sinus sampling, and even pituitary surgery.

**Case Report:**

A 41-year-old male with a history of right open adrenalectomy presented for evaluation of recent-onset abdominal distension, weight gain, facial fullness, and fatigue. He also had newly diagnosed diabetes and hypertension. Physical examination revealed facial fullness with plethora, centripetal obesity, and dilated abdominal veins. Biochemical evaluation revealed endogenous Cushing syndrome (late-night serum cortisol of 30.9 μg/dL, late-night salivary cortisol of 3.42 μg/dL, and overnight dexamethasone suppression test cortisol of 30.5 μg/dL), with hypokalemia (3.2 mmol/L) and grossly elevated dehydroepiandrosterone sulphate (2,857 μg/dL). Plasma ACTH as measured by the Immulite 1000 was unsuppressed (17.2 pg/mL) in the face of hypercortisolism, indicating ACTH dependence. Because of the patient's history and the known positive bias of plasma ACTH measurements from the immunoassay platform used for the assay, abdominal and thoracic imaging was performed, revealing multiple metastatic deposits in the liver and lungs. The left adrenal gland was atrophic, confirming that ACTH was truly suppressed. A diagnosis of metastatic adrenocortical carcinoma with adrenal Cushing syndrome was considered, and a chemotherapy regimen comprising etoposide, doxorubicin, and cisplatin was initiated. Ketoconazole was added later to control hypercortisolism. The patient took ketoconazole for 1 month and was then lost to follow-up.

**Conclusion:**

Spurious plasma ACTH measurements can occur with the Immulite 1000 and falsely indicate ACTH-dependent Cushing syndrome. Consequently, the clinical context must be carefully considered when evaluating patients with hypothalamic-pituitary-adrenal axis disorders.

## INTRODUCTION

Endogenous Cushing syndrome is a rare disorder, with a reported incidence of 3.2 cases per million persons per year.^1^ The condition is caused by excessive cortisol production by the adrenal glands and is characterized by the loss of circadian rhythm of cortisol secretion and the negative feedback regulation of the hypothalamic-pituitary-adrenal axis. Symptoms stem from high cortisol levels and include weight gain (especially in the centripetal areas), fatigue, proximal muscle weakness, thin skin, stretch marks, easy bruising, fractures, hirsutism, and irregular menses.^2^

Endogenous Cushing syndrome is broadly subtyped into adrenocorticotropic hormone (ACTH)–independent (adrenal source) and ACTH-dependent (pituitary or ectopic source) types based on morning (0800 h-1000 h) ACTH measurement.^3^ A suppressed plasma ACTH level (<5 pg/mL) suggests the ACTH-independent type, while an elevated level (>15 pg/mL) suggests the ACTH-dependent type.^2^ Plasma ACTH levels between 5 and 15 pg/mL are considered indeterminate.^2^ The ACTH-dependent type accounts for 70% to 80% of all cases of endogenous Cushing syndrome, while the remaining 20% to 30% of cases are the ACTH-independent type.^4^ Among the cases of ACTH-independent Cushing syndrome, adrenal adenoma accounts for 70% to 80% of cases, and adrenocortical carcinoma accounts for 20% to 30% of cases.^4^

ACTH is typically measured using a 2-site immunometric (or sandwich) immunoassay. The assay utilizes a solid phase or capture antibody and a label or detector antibody that are directed against the 2 nonoverlapping epitopes of the sandwiched antigen. A positive ACTH bias has been reported with a certain immunoassay platform (Immulite 1000 [Siemens AG]) that can lead to a false interpretation of ACTH-independent Cushing syndrome as ACTH-dependent Cushing syndrome and a false interpretation of secondary adrenal insufficiency as primary adrenal insufficiency.^5-7^ Awareness of this platform-specific issue is important to direct correct diagnosis and management and prevent unnecessary investigations and interventions.

We report a case in which a spurious plasma ACTH elevation was suspected and appropriately managed in a patient with metastatic adrenocortical carcinoma.

## CASE REPORT

A 41-year-old male presented to our hospital in 2024 with symptoms of abdominal distention, weight gain, facial puffiness, and fatigue for the prior 2 months. He had been diagnosed with systemic hypertension and diabetes mellitus 1 month prior. The patient's history was noteworthy for acute-onset right hypochondriac pain in 2020 that was managed at another hospital. Contrast-enhanced computed tomography (CT) of the abdomen and thorax performed at that time revealed a 9.2 × 9.5 × 7.4-cm mass in the right suprarenal region and a 2.3 × 1.8-cm nodular lesion in the posterior-basal segment of the lower lobe of the right lung. The patient underwent right open adrenalectomy (no hormonal details available), and the histopathology report suggested a moderately differentiated grade 2 adrenocortical carcinoma. CT-guided fine needle aspiration cytology from the lung lesion indicated a metastatic deposit. However, no further intervention was performed, and the patient was lost to follow-up until the presentation to our hospital in 2024.

On physical examination, the patient's height was 177.8 cm, weight was 81.0 kg, body mass index was 25.62 kg/m^2^, and blood pressure was 156/94 mm Hg. The patient had facial fullness with plethora, centripetal obesity, and dilated abdominal veins. No striae or ecchymotic patches, proximal muscle weakness, spine tenderness or deformity, or hyperpigmentation was noted.

Laboratory investigations showed elevated levels of 0800 h serum cortisol, 2300 h serum cortisol, and 2300 h salivary cortisol, as well as unsuppressed overnight dexamethasone suppression test cortisol, indicating endogenous Cushing syndrome (Table 1). Dehydroepiandrosterone sulphate (DHEAS) was elevated. A normal serum testosterone level with suppressed gonadotropins indicated an adrenal source of androgen production. Hypokalemia and a low-renin, low-aldosterone state were also documented, suggesting hypercortisolism-mediated hypokalemia (Table 1). The patient's 0800 h plasma ACTH as measured by the Immulite 1000 was 17.2 pg/mL, indicating an ACTH-dependent cause of endogenous Cushing syndrome (Table 1). However, given the patient's history of partially treated adrenocortical carcinoma and a literature review suggesting platform-specific issues, ACTH-independent Cushing syndrome was suspected, and a contrast-enhanced CT of the abdomen was performed.

**Table 1.** Patient's Initial Laboratory and Hormonal Parameters

Parameter	Value	Reference Range/Remarks
Hemoglobin, g/dL	13.8	13.2-16.6
Urea, mg/dL / Creatinine, mg/dL	30 / 0.93	20-40 / 0.6-1.2
Sodium, mmol/L / Potassium, mmol/L	135 / 3.2	135-145 / 3.5-5.5
Serum glutamic-oxaloacetic transaminase, IU/L / Serum glutamic-pyruvic transaminase, IU/L	238 / 52	<40 / <40
Alkaline phosphatase, IU/L	293	30-120
Albumin, g/dL	3.48	3.5-5.5
HbA1c, %	7.4	<5.7
Serum cortisol at 0800 h, μg/dL	39.3	3.7-19.4
Serum cortisol at 2300 h, μg/dL	30.9	<7.5
Salivary cortisol at 2300 h, μg/dL	3.42	<0.243
Overnight dexamethasone suppression test cortisol, μg/dL	30.5	<1.8
Plasma adrenocorticotrophic hormone (ACTH) at 0800 h, pg/mL	17.2	<5: ACTH-independent Cushing syndrome >15: ACTH-dependent Cushing syndrome
Plasma aldosterone concentration, ng/dL / Direct renin concentration, mIU/L	4.56 / 5.19	Suggestive of low-renin, low-aldosterone hypertension
Dehydroepiandrosterone sulphate, μg/dL	2,857	140-484
17-hydroxyprogesterone, ng/mL	10.3	<1.5
Testosterone, ng/dL	672.9	240-871
Luteinizing hormone, IU/L / Follicle stimulating hormone, IU/L	0.16 / 0.14	0.6-12.0 / 1.0-12.0

**Figure 1.**
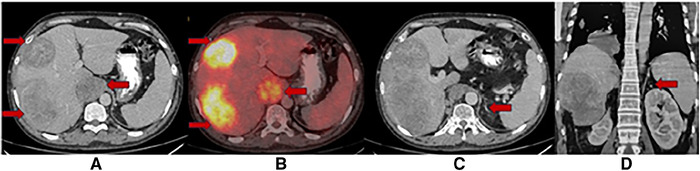
(A) Computed tomography (CT) axial image of the abdomen shows multiple liver metastases (arrows). (B) 18F-fluorodeoxyglucose (FDG) positron emission tomography/CT axial image of the abdomen shows multiple FDG-avid liver metastases (arrows). (C) CT axial image of the abdomen shows atrophied left adrenal gland (arrow). (D) CT coronal image of the abdomen shows atrophied left adrenal gland (arrow).

**Figure 2.**
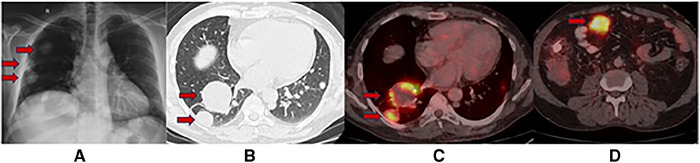
(A) Chest radiograph posteroanterior view shows multiple lung metastases (arrows). (B) Computed tomography (CT) axial image of the thorax shows multiple lung metastases (arrows). (C) 18F-fluorodeoxyglucose (FDG) positron emission tomography (PET)/CT axial image of the thorax shows multiple FDG-avid lung metastases (arrows). (D) 18F-FDG PET/CT axial image of the abdomen shows FDG-avid peritoneal metastasis (arrow).

Imaging revealed large, ill-defined, heterogeneously enhancing hypodense lesions in the liver, predominantly involving segments VI and VII and the caudate lobe, and obliterating the lumen of the hepatic portion of the inferior vena cava. The left adrenal gland was atrophic, reinforcing that the metastatic lesions produced excess cortisol and suppressed pituitary ACTH, thereby causing the left adrenal gland to atrophy (Figure 1). 18F-fluorodeoxyglucose positron emission tomography/CT showed multiple tracer-avid metastatic lesions in the lung, liver, and peritoneum (Figures 1 and 2). Based on the patient's history, clinical findings, and imaging, a diagnosis of metastatic adrenocortical carcinoma with ACTH-independent (adrenal) Cushing syndrome and spurious ACTH elevation was considered.

The patient was initiated on spironolactone (100 mg orally twice daily) and cilnidipine (10 mg orally twice daily) for hypertension and was administered multiple doses of insulin for diabetes (regular insulin 3 U subcutaneous before each meal and neutral protamine Hagedorn insulin 5 U subcutaneous at bedtime). Following medical oncology consultation, the patient was treated with 6 cycles of chemotherapy comprising etoposide, doxorubicin, and cisplatin (doxorubicin 60 mg intravenous [IV] on day 1, etoposide 150 mg IV on days 2 to 4, and cisplatin 60 mg IV on days 3 and 4).

Postchemotherapy follow-up evaluation (6 months from the patient's initial presentation at our hospital) revealed a reduction in the size of the metastatic lesions, with persisting hypercortisolism (Table 2). Ketoconazole 100 mg orally 3 times daily was added to the patient's treatment regimen. The patient took ketoconazole for 1 month and was lost to further follow-up.

**Table 2.** Patient's Postchemotherapy Laboratory and Hormonal Parameters

Parameter	Value	Reference Range/Remarks
Hemoglobin, g/dL	12.1	13.2-16.6
Urea, mg/dL / Creatinine, mg/dL	23 / 0.85	20-40 / 0.6-1.2
Sodium, mmol/L / Potassium, mmol/L	140 / 4.3	135-145 / 3.5-5.5
Serum glutamic-oxaloacetic transaminase, IU/L / Serum glutamic-pyruvic transaminase, IU/L	31 / 35	<40 / <40
Alkaline phosphatase, IU/L	312	30-120
Albumin, g/dL	4.25	3.5-5.5
HbA1c, %	6.6	<5.7
Serum cortisol at 0800 h, μg/dL	20.5	3.7-19.4
Overnight dexamethasone suppression test cortisol, μg/dL	29	<1.8
Plasma adrenocorticotrophic hormone (ACTH) at 0800 h, pg/mL	9.26	<5: ACTH-independent Cushing syndrome >15: ACTH-dependent Cushing syndrome
Testosterone, ng/dL	515.61	240-871

## DISCUSSION

Lila et al recommended taking a stepwise investigative approach for a patient with suspected Cushing syndrome.^8^ The first determination is whether the patient has endogenous Cushing syndrome. Our patient had elevated morning cortisol, evidence of abnormal cortisol diurnal rhythm, and impaired feedback regulation, confirming endogenous Cushing syndrome. The next determination is the cause of endogenous Cushing syndrome. As noted previously, morning plasma ACTH levels broadly subtype endogenous Cushing syndrome as ACTH-independent (<5 pg/mL), ACTH-dependent (>15 pg/mL), or indeterminate (5-15 pg/mL).^2^ ACTH is a heat-labile peptide; the sample should be collected in prechilled EDTA tubes, and a cold chain should be maintained during sample transportation.^9^ A suppressed plasma ACTH (<5 pg/mL) value should be confirmed by repeated careful measurements to exclude a false low value related to preanalytical errors.

In our patient, the plasma ACTH level was inappropriately high at 17.2 pg/mL, contrary to the clinical scenario. Discordant ACTH levels in patients with adrenal Cushing syndrome have been previously reported.^5-7^ In the Gosavi et al case series on adrenal Cushing syndrome, the authors reported that 9 of 58 patients had unsuppressed ACTH levels (>20 pg/mL) according to assays performed on the Siemens Immulite platform.^5^ The patients’ ACTH levels ranged from 25.4 pg/mL to 52.9 pg/mL, and the final diagnosis was adrenocortical carcinoma in 8 patients and adrenocortical adenoma in 1 patient. ACTH assays were repeated on the Liaison platform (Diasorin S.p.A.) for 5 patients, and the levels were found to be appropriately low for the clinical scenario. The authors suggested caution when interpreting ACTH results from the Immulite platform, especially in adrenocortical carcinoma cases, and recommended the use of other platforms.^5^

Greene et al reported 5 cases in which falsely elevated ACTH results from the Siemens Immulite platform confounded the diagnosis and differential diagnosis of Cushing syndrome; the discrepancy was resolved when the assay was repeated using other platforms: the Roche cobas (cobas solutions by Roche Diagnostics, North America) and the AIA (Tosoh Bioscience, Inc).^6^ As shown in the Greene et al case series, spurious ACTH elevation can lead to unnecessary investigations and interventions. One patient in their series underwent pituitary imaging, inferior petrosal sinus sampling, and pituitary surgery. On follow-up, the patient was determined to not have Cushing syndrome.^6^

DHEAS level has been suggested as a surrogate marker to differentiate between ACTH-dependent and ACTH-independent Cushing syndrome.^10^ Unlike adrenocortical adenomas, adrenocortical carcinomas frequently co-secrete adrenal androgens along with cortisol, and DHEAS levels may not be discriminatory. However, a very high DHEAS level, as seen in our case, favors a diagnosis of adrenocortical carcinoma.^11^

Several reasons have been postulated for spuriously high ACTH levels, one of them being assay interference. The Siemens Immulite 1000 may be susceptible to interference by heterophile antibodies that can bridge the detection and capture antibodies in the absence of antigen and result in falsely high ACTH levels. This interference can be mitigated by (a) using an alternative analytical platform, (b) demonstrating nonlinearity of results on serial dilution, (c) employing polyethylene glycol precipitation, or (d) using heterophile blocking reagents.^6,12,13^

Another reason for spuriously high ACTH levels could be incorrect measurement of proopiomelanocortin and ACTH fragments (as ACTH) with relatively less specific polyclonal antibodies.^6^ Shi et al reported that for discordant immunoassay samples between the Elecsys (Roche Diagnostics, North America) and Siemens Immulite 1000 platforms, only the Elecsys ACTH results correlated with liquid chromatography tandem mass spectrometry–intact ACTH results.^14^

The problem of ACTH discrepancy is not limited to endogenous Cushing syndrome but has also been reported in the setting of adrenal insufficiency. Singha et al evaluated 104 patients with hemoglobin E/beta-thalassemia, with plasma ACTH measured by the Siemens Immulite 1000 and the Roche Elecsys platforms. The authors reported a significant positive bias with the Immulite 1000 (mean plasma ACTH of 83.4 pg/mL vs 52.1 pg/mL); furthermore, 2 patients with secondary adrenal insufficiency had falsely high ACTH levels, leading to potential misdiagnosis as primary adrenal insufficiency.^7^

Adrenocortical carcinoma is a rare malignancy with a reported incidence of 1.02 per million persons per year^15^ and an aggressive course, with an overall 5-year survival of 54% (ranging from 71% for stage I to 13% for stage IV disease).^16^ Ayala-Ramirez et al reported hormonal excess in 42% of adrenocortical carcinoma cases; cortisol excess was the most common and accounted for 55% of all functional adrenocortical carcinomas.^17^ Endogenous Cushing syndrome from functioning metastatic lesions can be a presenting feature of adrenocortical carcinoma recurrence.^18^ Although an initial hormonal workup was not available for our patient, the clinical presentation suggested that hypercortisolism was of recent onset and resulted from cortisol hypersecretion from the metastatic deposits. Notably, metastatic lesions manifesting 4 years after the initial presentation retained functional steroidogenesis and manifested as autonomous cortisol and DHEAS excess.

Chemotherapy is the principal modality for the management of metastatic adrenocortical carcinoma.^19^ Considering our patient's profile, mitotane would have been an ideal option; however, this agent is not available in our country. A combination chemotherapy comprising etoposide, doxorubicin, and cisplatin was used instead. This regimen has been added to the standard of care for metastatic adrenocortical carcinoma following the FIRM-ACT trial that demonstrated better tumor response and progression-free survival with etoposide, doxorubicin, and cisplatin plus mitotane compared to streptozocin plus mitotane.^20^

## CONCLUSION

A spurious elevated plasma ACTH level obtained via Immulite 1000 assay could have led to a potential misdiagnosis of ACTH-dependent Cushing syndrome in a patient with metastatic adrenocortical carcinoma. The clinical context must be carefully considered when evaluating patients with hypothalamic-pituitary-adrenal axis disorders.

## Notes

*This article meets the Accreditation Council for Graduate Medical Education and the American Board of Medical Specialties Maintenance of Certification competencies for Patient Care and Medical Knowledge.*
